# Comparative Efficacy and Safety of Intralesional MMR Vaccine and Vitamin D3 in Managing Nongenital Warts: A Systematic Review and Meta‐Analysis

**DOI:** 10.1111/jocd.70618

**Published:** 2026-01-08

**Authors:** Rahul Balach, Ayan Khalid, Anas Rasool, Aamna Kashif, Abdullah Ahmad, Aroosa Zafar, Muhammad Ibrahim, Aamir Shahid Javed, Misha Hasan, Somaiya Ahmed

**Affiliations:** ^1^ Ziauddin University Karachi Pakistan; ^2^ Dow University of Health Sciences Karachi Pakistan; ^3^ Faisalabad Medical University Faisalabad Pakistan; ^4^ CMH Lahore Medical College Lahore Pakistan; ^5^ POF Hospital, Wah Medical College Wah Cantt Pakistan; ^6^ Sir Salimullah Medical College Dhaka Bangladesh

**Keywords:** cutaneous warts, human papillomavirus, intralesional immunotherapy, meta‐analysis, MMR vaccine, randomized controlled trials, vitamin D3

## Abstract

**Background:**

Cutaneous warts are common benign lesions caused by human papillomavirus and often resist conventional treatments. Intralesional immunotherapy with measles–mumps–rubella (MMR) vaccine or vitamin D3 has emerged as an alternative, but their comparative efficacy and safety remain unclear.

**Methods:**

We conducted a systematic review and meta‐analysis of nine randomized controlled trials (RCTs) including 742 patients comparing intralesional MMR with vitamin D3 for nongenital cutaneous warts. We searched PubMed, Cochrane CENTRAL, and ScienceDirect up to June 2025, and the protocol was registered in PROSPERO (ID: 1128443). Primary outcomes were complete, partial, and no response; secondary outcomes were recurrence and adverse events (erythema, pain, swelling). Pooled risk ratios (RRs) with 95% confidence intervals (CIs) were calculated.

**Results:**

Vitamin D3 achieved lower complete clearance than MMR (RR 0.85; 95% CI 0.75–0.96; *p* = 0.01) and a higher risk of no response (RR 2.40; 95% CI 1.40–4.13; *p* = 0.002). No significant differences were seen for partial response (RR 1.24; 95% CI 0.94–1.64; *p* = 0.13; *I* ^2^ = 0%), recurrence (RR 2.20; 95% CI 0.73–6.58; *p* = 0.16), or pain (RR 0.92; 95% CI 0.74–1.14; *p* = 0.46). Vitamin D3 was linked to more swelling (RR 2.79; 95% CI 1.15–6.75; *p* = 0.02), while MMR was associated with more erythema (RR 0.60; 95% CI 0.42–0.86; *p* = 0.006).

**Conclusions:**

Intralesional MMR appears to be more effective than vitamin D3 for cutaneous warts, with superior clearance and distinct adverse event profiles. Larger, standardized trials are warranted to validate these effect sizes and optimize treatment strategies.

## 1 Introduction

The human papillomavirus (HPV), particularly types 1, 2, 3, and 4, is the leading cause of non‐genital cutaneous warts, with plantar warts having an annual incidence of 14% [1]. While these lesions can often regress on their own, sometimes they may persist for years and significantly affect a person's quality of life [2, 3]. Due to the discomfort, potential scarring, and high recurrence rates associated with traditional treatments like cryotherapy, salicylic acid, and electrocautery, there has been growing interest in immunomodulatory treatments [4].

A key factor in wart persistence is the virus's ability to evade host immunity by inhibiting antigen presentation and local immune responses [5]. To counter this, several intralesional immunotherapies have gained attention as an effective option for treating non‐genital viral warts, as it can trigger systemic immune responses that promote the clearance of both treated and distant lesions. These include the measles–mumps–rubella (MMR) vaccine, purified protein derivative (PPD), interferons, imiquimod, and vitamin D3. Among these, MMR and vitamin D3 have attracted the most attention due to their accessibility, low cost, and promising clearance rates. With a recent review by, Mullen reporting complete response rates of 27%–90% for MMR and 40%–96% for vitamin D3 [6].

MMR and vitamin D3 have different mechanisms of action. The MMR vaccine enhances the systemic elimination of HPV‐infected skin cells by inducing a Th1‐mediated immune response. In addition, intralesional MMR promotes mononuclear cell proliferation and triggers the release of cytokines such as interleukin‐2 (IL‐2), tumor necrosis factor (TNF), and interferons (IFNs), which further activate cytotoxic T lymphocytes and natural killer cells involved in clearing HPV‐infected tissues [7, 8]. Whereas vitamin D3 supports keratinocyte development and increases the production of antimicrobial peptides, which assist in clearing the infection by modulating both innate and adaptive immunity [5, 9, 10]. Moreover, activation of toll‐like receptors (TLRs) on macrophages and keratinocytes upregulates VDR and vitamin D–activating enzymes, thereby enhancing the synthesis of antimicrobial peptides, such as defensin β2 and cathelicidin. Through vitamin D receptor (VDR)–dependent signaling, vitamin D3 suppresses key pro‐inflammatory cytokines such as interleukin‐6 (IL‐6), interleukin‐8 (IL‐8), tumor necrosis factor‐alpha (TNF‐α), and interferon‐gamma (IFN‐γ), thereby promoting an anti‐inflammatory and regulatory immune profile [11].

Beyond MMR and vitamin_3_, recent studies continue to explore other intralesional immunogens. In 2022, Nofal et al. reported that complete clearance was achieved in 82.5% of patients treated with Candida antigen, and 55.6% with PPD [12]. Furthermore, in 2018, Salman et al. conducted a meta‐analysis of 17 randomized controlled trials comparing intralesional immunotherapy with cryotherapy, placebo, and imiquimod. They found that PPD and MMR were the most effective agents in achieving complete clearance, and intralesional immunotherapy was generally associated with fewer adverse effects compared to conventional treatments for non‐genital warts [13]. Although high clearance rates were reported with Candida antigen, MMR, PPD, and MIP, the efficacy of vitamin D3 was comparatively lower. A recent 2024 study by Sallam et al. reported higher complete clearance with intralesional MMR (80.4%) compared to vitamin D3 (66.1%) in patients with multiple warts. Regarding adverse effects, MMR was associated with higher rates of mild pain (96.4%) and injection‐site itching (12.5%) than vitamin D3, which was generally well tolerated. Although MMR has been used longer and is supported by more comparative studies, vitamin D3 offers practical advantages, including lower cost, no cold chain requirement, easier availability, and use in immunocompromised patients [14]. Despite many studies having evaluated the efficacy of these agents individually and comparatively, findings remain inconsistent and no clear consensus has been reached on the superior treatment. Previous reviews have assessed these agents individually, but no meta‐analysis has directly compared their efficacy and safety. Considering the global burden of warts and variability in outcomes, an evidence‐based comparison is needed. This meta‐analysis aims to analyze existing evidence and compare the efficacy of the intralesional MMR vaccine and vitamin D3 in treating non‐genital warts, thereby informing clinical practice and guiding future therapeutic strategies.

## 2 Methods

This systematic review and meta‐analysis was conducted in accordance with the Cochrane Handbook for Systematic Reviews of Interventions and reported following the Preferred Reporting Items for Systematic Reviews and Meta‐Analyses (PRISMA) guidelines (Table S4) [15, 16]. All methods were specified a priori, and the study protocol was registered prospectively in the International Prospective Register of Systematic Reviews (PROSPERO, ID: 1128443).

### 2.1 Eligibility Criteria

Trials that met the following inclusion criteria were eligible: (1) randomized controlled trials (RCTs); (2) studies enrolling immunocompetent patients aged ≥ 4 years; (3) participants with cutaneous, non‐genital warts, specifically including verruca vulgaris, palmoplantar warts, and verruca plana; and (4) trials directly comparing intralesional measles–mumps–rubella (MMR) vaccine with intralesional Vitamin D3.

Exclusion criteria were: (1) studies exclusively conducted on genital warts; (2) single‐arm studies; and (3) case reports, reviews, meta‐analyses, or observational studies without a comparator arm.

### 2.2 Search Strategy and Study Selection

A comprehensive electronic search of PubMed, Cochrane Central Register of Controlled Trials, and ScienceDirect was conducted to identify eligible studies published up to June 2025 in any language using the following search terms: (“cutaneous warts” OR “verruca vulgaris” OR “common warts” OR “warts” OR “verrucae” OR “skin warts”) AND ((“Vitamin D” OR “cholecalciferol” OR “ergocalciferol” OR “calcitriol” OR “vitamin D3” OR “vitamin D2” OR “intralesional vitamin D”) OR (“MMR vaccine” OR “measles‐mumps‐rubella vaccine” OR “intralesional MMR” OR “intralesional immunotherapy” OR “MMR injection”)) AND (“therapeutics” OR “therapy” OR “treatment” OR “therapeutic use” OR “comparative study” OR “randomized controlled trial” OR “clinical trial”). Additionally, reference lists of retrieved articles and previous reviews were screened for potentially eligible trials.

All records were imported into Rayyan.ai [17], and duplicates were removed. The remaining articles were screened in two stages: title/abstract screening followed by full‐text assessment. Two reviewers independently performed the selection process, and discrepancies were resolved through discussion with a third author until consensus was achieved.

### 2.3 Endpoints

The primary endpoint was the response in injected warts, categorized as complete (defined as clearance of 100% of warts), partial (< 100% clearance), or no response.

Secondary endpoints included recurrence of warts and treatment‐related adverse events, such as pain, erythema, and swelling.

### 2.4 Data Extraction and Quality Assessment

Trial characteristics, baseline demographics, intervention details, and outcome data were extracted into a predesigned Microsoft Excel spreadsheet. Risk of bias for each included RCT was assessed using the Cochrane Risk of Bias tool [18], evaluating the following domains: sequence generation, allocation concealment, blinding, incomplete outcome data, selective reporting, and other biases. Each domain was classified as low risk, high risk, or some concerns.

Data extraction and risk‐of‐bias assessment were independently conducted by two reviewers. Any differences were resolved through discussion, and if needed, by involving a third reviewer to reach consensus.

### 2.5 Statistical Analysis

All analyses were performed using Review Manager (Version 5.4.1, Copenhagen: The Nordic Cochrane Center, The Cochrane Collaboration). Dichotomous outcomes were analyzed using the Cochran–Mantel–Haenszel method, and effect sizes were pooled to calculate risk ratios (RRs) and corresponding 95% confidence intervals (CIs). A random‐effects model was used for all meta‐analyses, consistent with Berkey et al. [19], to account for anticipated clinical and methodological variability across included trials. Forest plots were used to display pooled results, while funnel plots were generated to explore potential publication bias (Figure S14).

Statistical heterogeneity was assessed using Higgins' *I* ^2^ statistic, with thresholds of 25%, 50%, and 75% representing low, moderate, and high heterogeneity, respectively [20]. The Chi‐square test was performed to assess differences between the subgroups. Subgroup analyses were performed to examine the effect of Vitamin D3 dosage per injection (< 100 000 IU vs. > 100 000 IU) on treatment efficacy, independent of MMR‐related variability.

Where significant heterogeneity was present, sensitivity analyses were performed by sequentially excluding each study to test the robustness of the findings. A two‐sided *p*‐value < 0.05 was considered statistically significant.

## 3 Results

### 3.1 Study Selection and Baseline Characteristics

**FIGURE 1:**
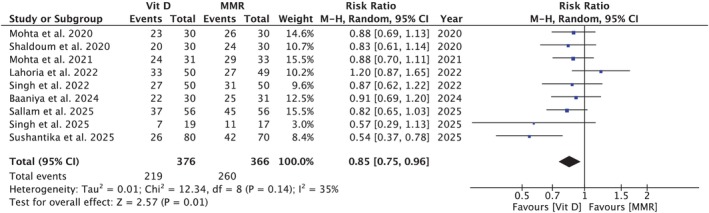
Forest plot comparing complete resolution rates between vitamin D3 and MMR. Vitamin D3 showed significantly higher complete‐resolution rates than MMR; *p* = 0.01.

The initial search yielded 382 results. After removal of duplicate records and ineligible studies, nine randomized controlled trials (RCTs) were included, comprising 742 patients (Figure S1) (Figure 1) [5, 6, 7, 8, 9, 10, 14, 21, 22, 23, 24, 25, 26, 27, 28, 29, 30, 31, 32, 33, 34, 35, 36, 37, 38, 39, 40, 41, 42]. Participants were predominantly adolescents and young adults, with mean ages ranging from 12.7 to 33.2 years. Mean wart duration ranged from 5.9 to 22.1 months across studies (Table 1). Verruca vulgaris and palmoplantar warts were most common, with smaller numbers of verruca plana, periungual, filiform, and genital warts (Table S1). Prior treatment was reported in four trials, with rates ranging from 4% to 67% of patients across studies, though recent therapy was generally an exclusion criterion (Table S2). Interventional protocols included intralesional Vitamin D3 injections and MMR vaccine immunotherapy, with variations in dose, frequency, and number of treatment sessions across trials (Table S3).

**TABLE 1:** Participant baseline characteristics.

Study	Shaldoum et al. [9]	Mohta et al. [40]	Mohta et al. [41]	Singh et al. [10]	Lahoria et al. [42]	Sushantika et al. [8]	Singh et al. [7]	Baaniya et al. [14]	Sallam et al. [5]
Participants in MMR vaccine arm, *n*	30	30	33	50	49	70	17	31	56
Participants in Vitamin D3 arm, *n*	30	30	31	50	50	80	19	30	56
Interventions and dose per injection site									
Maximum treatment sessions, *n*	6	3	4	3	—	6	3	5	5
Dosing interval, weeks	3	4	2	2	2	3	3	3	2
MMR vaccine brand, manufacturer, country	VACSERA, Egypt	—	TRESIVAC, Serum Institute of India Ltd., Pune, Maharashtra, India	—	—	TRESIVAC, Serum Institute of India Ltd., Pune, Maharashtra, India	TRESIVAC, Serum Institute of India Ltd., Pune, Maharashtra, India	—	VACSERA, Egypt
Vitamin D3 type	MPCI.CA, Egypt	—	Intralesional Vitamin D3 or ARACHITOL‐6 L, Abbott, India	—	—	ARACHITOL‐6 L, Abbott, India	ARACHITOL‐6 L, Abbott, India	—	MCPI.CA, Egypt
Dose injected per site in MMR group, mL	0.3 into largest wart	0.1 into largest wart	0.2–0.3 into largest wart	0.3 into largest wart	0.5 into largest wart	0.3 + 0.1 into largest wart + other warts	0.5 into largest wart	—	0.3 into largest wart
Dose injected per site in Vitamin D3 group, mL	0.4 into wart base	0.1 into the largest wart	0.2–0.3 into the largest wart	0.2 into the largest wart	0.2 into the largest wart	0.2–0.5 into wart base	0.5 into the largest wart	0.5 intralesional	0.3 into the largest wart
Vitamin D3 IU per site; concentration^a^	4000; 10 000 IU/mL (2.5 mg/ml)	60 000; 600 000 IU/mL (15 mg/mL)	120 000–180 000; 600 000 IU/mL (15 mg/ml)	—	—	120 000–300 000; 600 000 IU/mL (15 mg/mL)	300 000; 600 000 IU/mL (15 mg/mL)	300 000; 600 000 IU/mL	1800; 6000 IU/mL
Duration of warts, months (mean ± SD)^b^									
MMR group	14.7 ± 14.5	6.8 ± 4.5	9.1 ± 6.0	22.1 ± 24.0	14.8 ± 20.5	—	6.8 ± 3.5	—	17.4 ± 9.2
Vitamin D3 group	13.2 ± 15.8	7.5 ± 4.6	8.4 ± 5.9	11.6 ± 14.2	8.6 ± 17.0	—	5.9 ± 4.1	—	21.2 ± 9.8
Outcomes in injected warts^c^									
Complete response, % (MMR/Vitamin D3)^d^	44 (80.0/66.7)	49 (86.7/76.7)	53 (87.8/77.4)	58 (62.0/54.0)	—	68 (60.0/32.5)	18 (64.1/36.8)	47 (80.6/73.4)	82 (80.4/66.1)
Partial response, % (MMR/Vitamin D3)^e^	12 (13.3/26.7)	5 (6.7/10.0)	7 (6.1/16.1)	27 (28.0/26.0)	—	66 (37.1/50.0)	13 (23.5/47.4)	12 (16.1/23.3)	12 (14.3/7.1)
No response, % (MMR/Vitamin D3)	4 (6.7/6.7)	6 (6.6/13.3)	4 (6.1/6.5)	15 (10.0/20.0)	0	16 (2.9/17.5)	2 (5.8/5.2)	2 (3.2/3.3)	18 (5.4/26.8)
Recurrence, % (MMR/Vitamin D3)^f^	0 (0.0/0.0)	0 (0.0/0.0)	0/0 (0.0/0.0)	—	3 (4.1/2.0)	—	3 (5.9/10.5)	4 (4.0/13.6)	5 (3.6/5.4)
Pain, % (MMR/Vitamin D3)	44 (80.0/66.7)	17 (26.7/30)	19 (27.3/32.3)	101 (100.0/100.0)	—	119 (60.0/95.0)	—	61 (100.0/100.0)	67 (100.0/19.6)
Swelling, % (MMR/Vitamin D3)	4 (0.0/13.3)	13 (16.7/26.7)	13 (15.2/25.8)	21 (8.0/34.0)	—	—	—	18 (0.0/60.0)	1 (1.7/0.0)
Erythema, % (MMR/Vitamin D3)	42 (100.0/40.0)	12 (16.7/23.3)	12 (15.2/22.6)	—	—	—	—	1 (0.0/3.3)	7 (10.7/1.8)

*Note:* Data are *n*, mean ± SD or *n* (%/%) rounded to one decimal place. — = not reported. All studies used MMR vaccine diluted in 0.5 mL distilled water (dilution information not reported in studies Singh et al. [10] and Lahoria et al. [42]). All IU/mL values calculated using 1 mg = 40 000 IU.

Abbreviation: IU, international units.

^a^ Maximum Vitamin D3 dose specified in Shaldoum et al. [9] was 5 mg per session. Studies Shaldoum et al. [9], Sushantika et al. [8] and Baaniya et al. [14] injected 5 warts per session.

^b^ Duration in Singh et al. [10] originally reported in weeks; converted to months using 1 month = 4.345 weeks.

^c^ Sushantika et al. [8] outcomes at 18 weeks reported above.

^d^ Lahoria et al. [42] reported complete response rates of 55% MMR, 66% vitamin D3, without providing absolute counts.

^e^ Data from Shaldoum et al. [9] showing minimal/partial responses to MMR (2/2) and Vitamin D3 (6/2) reported combined in the table as partial response. Similarly, data from Singh et al. [7] showing minimal/partial responses to MMR (2/2) and Vitamin D3 (3/6) and data from Baaniya et al. [14] showing excellent/good response to MMR (2/3) and Vitamin D3 (6/1) also reported combined as partial response.

^f^ Mohta et al. [40, 41] both reported no distal‐wart recurrences in MMR vaccine and 2 recurrences with Vitamin D3. Sushantika et al. [8] noted brief erythema and swelling, slightly higher with Vitamin D3; similar findings, which also included pain noted in Lahoria et al. (52%). Singh et al. [7] reported side effects like pain and swelling in 3 MMR patients vs 10 Vitamin D3 patients, without separate reporting.

Study quality varied, with risk of bias rated low in two trials, high in two, and raising some concerns in five (Figure S2).

### 3.2 Pooled Analysis of All Studies

#### 3.2.1 Complete Response in Injected Warts

A meta‐analysis of nine studies [5, 7, 8, 9, 10, 14, 40, 41, 42] including 376 participants in the Vitamin D3 group and 366 in the MMR group, demonstrated a statistically significant difference in complete response for injected warts, favoring the MMR group (RR = 0.85 [0.75, 0.96]; *p* = 0.01; *I* ^2^ = 35%; Figure 1). Subgroup analysis did not demonstrate effect modification by dosage per injection (*p*‐interaction: 0.45; Figure S3). Sensitivity analysis excluding the study by Sushantika et al. reduced heterogeneity to 0%, while the effect size remained consistent (RR = 0.89 [0.80, 0.98]; *p* = 0.02; *I* ^2^ = 0%; Figure S10).

#### 3.2.2 Partial Response in Injected Warts

**FIGURE 2:**
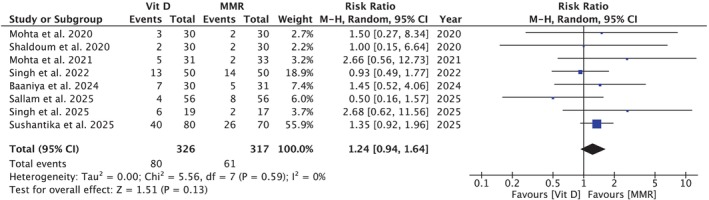
Forest plot comparing partial response between vitamin D3 and MMR. No significant difference was observed between the two groups; *p* = 0.13.

Seven studies [5, 7, 8, 9, 10, 14, 40, 41] including 326 participants in the Vit D group and 317 in the MMR group showed no statistically significant difference in partial response (RR = 1.24 [0.94, 1.64]; *p* = 0.13; *I* ^2^ = 0%; Figure 2). Subgroup analysis did not demonstrate effect modification by dosage per injection (*p*‐interaction: 0.16; Figure S4).

#### 3.2.3 No Response in Injected Warts

**FIGURE 3:**
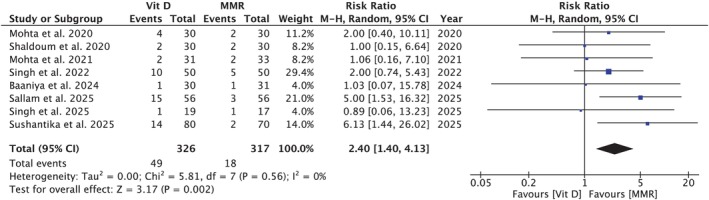
Forest plot comparing no‐response rates between vitamin D3 and MMR. Vitamin D3 was associated with significantly higher no‐response rates than MMR; *p* = 0.002.

Seven studies [5, 7, 8, 9, 10, 14, 40, 41] comprising 326 participants in the Vitamin D3 group and 317 in the MMR group found that Vitamin D3 was associated with a significantly higher risk of no response compared to MMR (RR = 2.40, 95% CI: 1.40, 4.13; *p* = 0.002; *I* ^2^ = 0%; Figure 3). Subgroup analysis did not demonstrate effect modification by dosage per injection (*p*‐interaction: 0.78; Figure S5).

#### 3.2.4 Recurrence of Warts

**FIGURE 4:**
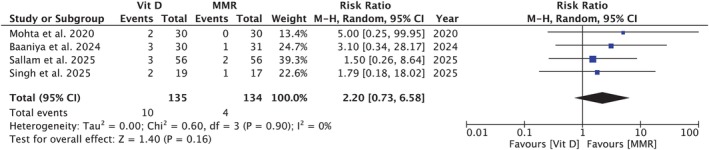
Forest plot comparing recurrence of warts between vitamin D3 and MMR. No significant difference was observed between the two groups; *p* = 0.16.

Four studies [5, 7, 14, 40], involving 135 participants in the Vit D3 group and 134 in the MMR group, showed no statistically significant difference in the recurrence of warts (RR = 2.20 [0.73, 6.58]; *p* = 0.16; *I* ^2^ = 0%; Figure 4). Subgroup analysis did not demonstrate effect modification by dosage per injection (*p*‐interaction: 0.89; Figure S6).

#### 3.2.5 Erythema

**FIGURE 5:**
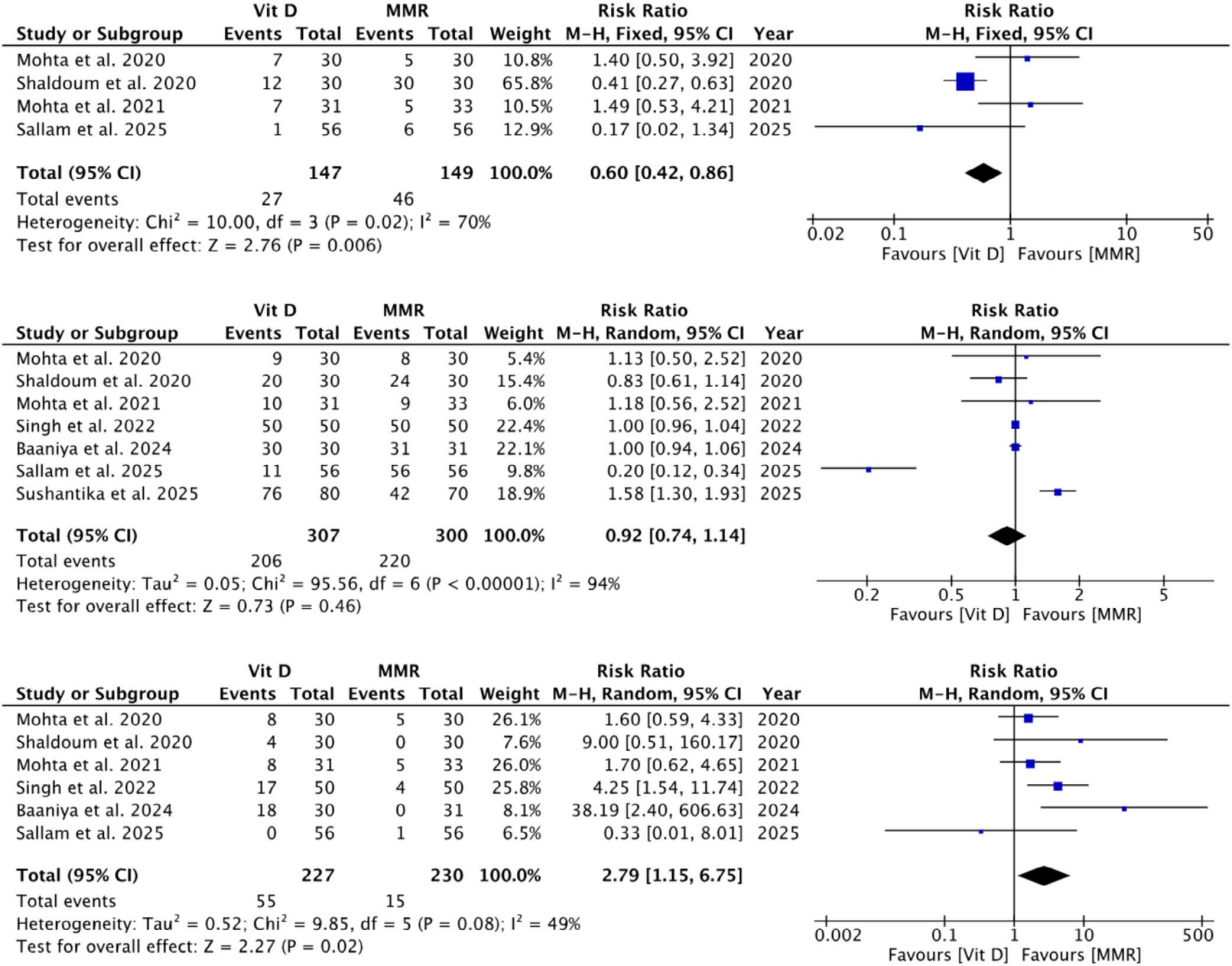
Adverse event forest plots for erythema, pain, and swelling. Forest plots comparing Vitamin D3 and MMR groups for erythema (A), pain (B), and swelling (C). Vitamin D3 showed significantly lower erythema rates compared with MMR (*p* = 0.006), no significant difference in pain incidence (*p* = 0.46), and higher swelling rates than MMR (*p* = 0.02).

Four studies [5, 9, 40, 41] with 147 participants in the Vit D3 group and 149 in the MMR group showed that Vit D3 was associated with a significantly lower risk of erythema compared to MMR group (RR = 0.60 [0.42, 0.86]; *p* = 0.006; *I* ^2^ = 70%; Figure 5). Subgroup analysis did not demonstrate effect modification by dosage per injection (*p*‐interaction: 0.17; Figure S7). Sensitivity analysis showed that exclusion of the study by Shaldoum et al. reduced the pooled effect estimate and heterogeneity (RR = 0.96 [0.50, 1.83]; *p* = 0.90, *I* ^2^ = 49%; Figure S11). Excluding any other study did not affect the effect size or heterogeneity.

#### 3.2.6 Pain

Six studies [5, 7, 8, 14, 40, 41] with 307 participants in the Vit D3 group and 300 in the MMR group found no statistically significant difference in pain between the groups (RR = 0.92 [0.74, 1.14]; *p* = 0.46; *I* ^2^ = 94%; Figure 5). Subgroup analysis did not demonstrate effect modification by dosage per injection (*p*‐interaction: 0.26; Figure S8). Sensitivity analysis reduced heterogeneity but didn't materially change the effect size (RR = 0.90 [0.69, 1.18]; *p* = 0.90, *I* ^2^ = 0%; Figure S12).

#### 3.2.7 Swelling

Five studies [5, 9, 10, 14, 40, 41] with 227 participants in the Vit D3 group and 230 in the MMR group showed that Vit D3 was associated with a significantly higher risk of swelling compared to MMR (RR = 2.79 [1.15, 6.75]; *p* = 0.02; *I* ^2^ = 49%; Figure 5). Subgroup analysis did not demonstrate effect modification by dosage per injection (*p*‐interaction: 0.49; Figure S9). Sensitivity analysis excluding the studies by Singh and Baaniya et al. reduced both heterogeneity and the effect size (RR = 1.69 [95% CI: 0.86, 3.30]; *p* = 0.13; *I* ^2^ = 0%; Figure S13).

## 4 Discussion

This meta‐analysis of nine studies compared the efficacy and safety of intralesional MMR vaccine and vitamin D3 for cutaneous warts. Several intralesional therapies have been explored for recalcitrant warts, including PPD, MIP, Candida antigen, and various pharmaceutical combinations, but there is no consensus on the most effective option. MMR, repurposed to trigger a systemic immune‐mediated attack, seems to achieve a higher complete clearance rates than vitamin D3, which acts locally by modulating keratinocyte growth and immune activity, while both demonstrated comparable recurrence but differing local side‐effect profiles.

Our analysis of the primary outcome showed that MMR appears more effective than vitamin D3 in achieving complete clearance of warts [5, 7, 14, 40, 41]. Sensitivity analysis reinforced the robustness of this effect: exclusion of the study by Sushantika et al. reduced heterogeneity to 0% while the effect size remained significant. Non‐response rates to intralesional therapy were generally higher with vitamin D3, suggesting a trend toward greater non‐responsiveness [8, 10, 14]. The apparent advantage of MMR may be related to its immunologic mechanism. By stimulating a Th1‐dominant delayed‐type hypersensitivity response against multiple antigens, the MMR vaccine promotes release of IL‐12, IL‐18, IFN‐γ, TNF‐α, and IL‐2, which activate cytotoxic T cells, natural killer cells, and macrophages while reducing IL‐10–mediated immunosuppression. This coordinated immune response can eliminate infected keratinocytes and, in some cases, lead to resolution of distant, uninjected lesions [3, 12, 43]. These mechanisms may partly explain the higher complete‐clearance rates observed with MMR. Clinically, MMR immunotherapy often achieves more rapid and widespread wart resolution, particularly in patients with multiple or recalcitrant lesions, whereas vitamin D3 typically demonstrates more modest, localized effects. MMR's more vigorous Th1‐mediated inflammation can also produce slightly higher rates of transient flu‐like symptoms, erythema, and swelling, although serious adverse events remain rare [44]. In contrast, partial response rates did not differ significantly between groups.

The proposed actions of vitamin D3 are less directly supported by wart‐specific studies and are largely inferred from broader immunology and dermatology research. Vitamin D3 binds to receptors on keratinocytes, where it can slow abnormal proliferation, promote differentiation, and enhance cutaneous innate immunity. Through activation of vitamin D response elements, it increases antimicrobial peptide production (e.g., cathelicidin LL‐37, human β‐defensin 2) and may also induce autophagy, facilitating viral degradation [11] Additional effects include activation of toll‐like receptors on keratinocytes and macrophages, leading to further antimicrobial peptide release and, in some cases, apoptosis of infected cells [4, 45, 46]. Overall, vitamin D3 produces a localized immune response that is modest but consistent, particularly in smaller or limited warts, with a generally mild side‐effect profile, mainly injection‐site pain, pruritus, dyspigmentation, or occasional vasovagal reactions. Because its activity is less systemic, distant‐lesion clearance is lower, and multiple treatment sessions may be required compared with MMR [47]. While these mechanisms provide a plausible basis for wart regression, the immune activation they generate is likely more localized compared to the systemic Th1‐mediated response induced by MMR. This difference may explain why vitamin D3 achieved partial regression of treated warts but showed relatively lower complete clearance rates compared with MMR. However, it should be noted that the included studies were generally small, geographically concentrated, and varied in injection protocols and outcome assessment, which may limit the generalizability of these findings.

These findings align with a 2022 systematic review and meta‐analysis by Ju et al., which reported that MMR achieved complete clearance in approximately 63% of injected lesions and 62% of distant lesions, whereas Vitamin D3 achieved complete clearance in around 54% of injected lesions but only 18% of distant lesions. This highlights the systemic immune‐stimulating effects of MMR compared with the primarily local action of Vitamin D3. Both therapies demonstrated low recurrence rates, around 2%, and were generally well tolerated. These results reinforce our conclusion that although both treatments are effective and safe, MMR may provide superior efficacy, particularly for patients with multiple or distant warts, due to its broader systemic immune activation [29].

Previous randomized trials of other intralesional immunotherapeutic agents, such as Candida antigen, and PPD, have also reported complete clearance rates in the range of 60%–80% for recalcitrant warts, with low recurrence and acceptable safety profiles [6]. Although these agents were not directly compared in our meta‐analysis, the efficacy of MMR and vitamin D3 appears broadly comparable to that of other intralesional immunotherapies, with MMR particularly favorable in patients with multiple or distant lesions. Our findings therefore support the growing view that host‐directed intralesional immunotherapy represents an effective alternative to destructive modalities for difficult warts.

In the study by Mohta et al., complete clearance rates varied according to wart type. For verruca vulgaris, Vitamin D3 achieved a slightly higher response (83.3%) compared to MMR (75%), though the difference was not statistically significant. In contrast, verruca plana responded significantly better to MMR (71.4%) than Vitamin D3 (22.2%), with *p* = 0.049 [41]. Overall, the data suggest that MMR may offer superior efficacy for verruca plana and palmoplantar warts, while Vitamin D3 can be equally effective, or slightly better, for verruca vulgaris. It is also worth noting that periungual warts were not consistently represented across all studies, limiting the ability to draw firm conclusions for this difficult‐to‐treat subtype. Future large‐scale, head‐to‐head randomized trials stratified by wart type and anatomical site are needed before firm conclusions can be drawn regarding the relative superiority of either intervention.

In our pooled analysis of four studies, we observed a trend toward reduced recurrence with MMR; however, this association did not reach statistical significance, indicating no definitive link and warranting cautious interpretation [2, 14, 48]. For instance, Sallam et al. reported recurrence rates of 3.6% in the MMR group versus 5.4% in the vitamin D3 group at 6 months of follow‐up, further highlighting the low but comparable recurrence across therapies [5]. Although the stronger cell‐mediated immune response elicited by MMR could theoretically contribute to more durable clearance compared with the more localized effects of vitamin D3, the current evidence remains inconclusive [49]. Larger, adequately powered trials are required before any firm conclusions can be drawn about differential recurrence risks between the two therapies.

Most participants in the included studies were immunocompetent adolescents and young adults, with only one trial conducted exclusively in a pediatric population [40]. This limits the extrapolation of findings to younger children, older adults, and immunocompromised patients, where immune responses and treatment tolerability may differ. Intralesional immunotherapy with MMR or vitamin D3 is generally considered a second‐line intervention, reserved for patients with recalcitrant or multiple warts who have failed first‐line therapies such as salicylic acid, cryotherapy, or electrosurgery. In this context, MMR immunotherapy represents a promising alternative, particularly in patients with multiple lesions or distant warts, as its systemic immune activation has the potential to achieve clearance of both treated and untreated sites.

Intralesional MMR vaccine and vitamin D3 provide practical, host‐directed options for patients with multiple or recalcitrant warts who have not responded to first‐line treatments. MMR is particularly suited for patients with numerous or distant lesions, whereas vitamin D3 offers a more localized approach suitable for smaller or limited warts. Typical treatment involves 2–5 sessions at 2–3 week intervals, and patient counseling should emphasize the gradual nature of wart regression. Incorporating these immunotherapies allows dermatologists and cosmetic physicians to expand their wart management strategies beyond destructive approaches, offering effective, immune‐based therapy with generally favorable cosmetic outcomes.

Our findings indicate that both intralesional MMR vaccine and vitamin D3 immunotherapy are generally safe, with adverse events that were almost exclusively mild, localized, and self‐limiting. The most common reactions across trials were transient swelling, erythema, and pain at the injection site, none of which required medical intervention or led to treatment discontinuation.

Both treatments caused local side effects, but overall, the MMR vaccine showed a more favorable safety profile, with fewer and milder reactions compared to vitamin D3 [41, 50, 51]. Several studies reported more frequent localized inflammatory reactions and swelling with vitamin D3 [7, 14, 51]. Although MMR is more immunogenic systemically, localized swelling primarily reflects injection‐site inflammation rather than overall immune potency. For example, Kavya et al. observed swelling in 78.6% of vitamin D3 recipients, all resolving within 4 weeks, while Baaniya et al. reported a median swelling duration of about 22 days [48]. In our pooled analysis of five studies, swelling was significantly higher with vitamin D3. Sensitivity analysis was performed due to moderate heterogeneity, sequential exclusion of two outlier studies [7, 14], which had high risk ratios strongly favoring vitamin D3, reduced heterogeneity to 0% while the effect estimate continued to favor MMR.

Compared to swelling, erythema showed an opposite trend [52]. Although some earlier studies found more erythema with vitamin D3, our analysis detected a higher rate with MMR [8, 51]. This was largely driven by Shaldoum et al. who reported erythema in all MMR‐treated patients [9]. Their protocol involved injecting only a single wart without local anesthesia, which likely provoked a more intense visible inflammatory response than in other studies. By contrast, vitamin D3 was typically administered as multiple dispersed injections and, in some trials, after topical anesthetic application, both of which can reduce immediate erythema. When the Shaldoum study was excluded in sensitivity analysis, heterogeneity dropped from 70% to 49% and the pooled effect became non‐significant, demonstrating that the apparent excess erythema with MMR was highly method dependent.

Pain was reported in both groups, indicating comparable pain incidence. Sensitivity analyses, excluding four studies, reduced heterogeneity but did not affect the overall effect size, confirming consistency. Importantly, no serious systemic adverse events were reported [7, 9, 14, 40]. Only a few trials monitored serum vitamin D and calcium, and one study noted biochemical hypervitaminosis in a small number of vitamin D3 patients, though symptomatic cases were rare [41]. Overall, both MMR and vitamin D3 immunotherapy were well tolerated, with side effects that were mild, local, and self‐limiting, consistent with prior evidence that systemic complications are uncommon.

Future research should address these gaps with large, multicenter randomized controlled trials that incorporate standardized dosing regimens, injection protocols, and follow‐up schedules. Trials should be stratified by wart type, anatomical site, and patient immune status to clarify whether particular subgroups derive differential benefit from MMR or vitamin D3. Rigorous safety monitoring, including biochemical assays for vitamin D and calcium, is needed to better characterize metabolic risks. Comparative studies evaluating combination strategies, longer‐term outcomes, cost‐effectiveness, and patient‐reported measures such as pain and quality of life would further inform clinical practice. Such high‐quality evidence will be essential to define the optimal role of intralesional MMR and vitamin D3 in the management of recalcitrant cutaneous warts.

## 5 Limitations and Conclusions

### 5.1 Limitations

The included studies were limited by small sample sizes having 742 patients, reducing statistical power for outcomes such as recurrence. Assessment of adverse reactions (e.g., erythema, pain) relied on either patient self‐report or clinician judgment without standardized scales, increasing the risk of measurement bias. Due to limitations in the available data, stratification by wart type was not feasible. Stratification by vitamin D3 dose per injection was performed using data from the vitamin D3 arm alone; this may not account for baseline differences in the MMR group, introducing potential misclassification. These subgroup results should therefore be interpreted with caution. Two of the included trials enrolled a small proportion of patients with genital warts, introducing potential clinical heterogeneity. Sensitivity analysis excluding these studies demonstrated that all primary and secondary efficacy outcomes remained consistent and materially unchanged. The only exception was adverse swelling, where statistical significance was lost upon excluding Singh et al., likely reflecting reduced statistical power, although the direction of effect continued to favor MMR. Collectively, these findings suggest that the inclusion of these trials did not influence the overall efficacy conclusions, while the effect on swelling should be interpreted as plausible but less precisely estimated. Additionally, procedural variations across studies may confound efficacy comparisons. Interventional protocols differed with respect to dose per injection, the number of lesions injected per session, treatment intervals, maximum number of sessions, and use of topical or local anesthesia, all of which may influence both perceived pain and visible inflammatory reactions. Outcome definitions and follow‐up durations for complete response, partial response, and recurrence were not fully uniform across trials, raising the possibility of outcome misclassification. Furthermore, seven of the nine trials were conducted in India and two in Egypt, so the evidence is derived from relatively homogeneous, single‐center studies in a limited number of countries, which may constrain generalizability to other geographic regions, ethnic groups, and health‐care settings. Finally Most studies reported short‐term follow‐up of 3–6 months, with only one study extending follow‐up beyond 12 months, highlighting a lack of long‐term data on treatment durability and recurrence.

### 5.2 Conclusion

This meta‐analysis demonstrates that the MMR vaccine appears to bemore effective than vitamin D3 for the treatment of cutaneous warts, achieving higher complete clearance rates and showing a trend toward more durable responses. Both therapies have generally favorable safety profiles, with differing patterns of local and systemic adverse events. Larger, multicenter randomized controlled trials with standardized dosing and follow‐up protocols are needed to confirm these findings and guide evidence‐based clinical decision‐making.

## Author Contributions

Rahul Balach: conceptualization, data curation, formal analysis, writing – original draft. Ayan Khalid: formal analysis, visualization, writing – original draft. Anas Rasool: data curation, software. Aamna Kashif: writing – original draft. Abdullah Ahmad: conceptualization, data curation. Aroosa Zafar: validation, writing – review and editing. Muhammad Ibrahim: visualization, writing – original draft. Aamir Shahid Javed: writing – review and editing. Misha Hasan: writing – review and editing. Somaiya Ahmed: conceptualization, supervision, writing – review and editing.

## Funding

The authors have nothing to report.

## Ethics Statement

The authors have nothing to report.

## Consent

The authors have nothing to report.

## Conflicts of Interest

The authors declare no conflicts of interest.

## Supporting information


**FIGURE S1:** PRISMA flow diagram outlining the literature search process.
**FIGURE S2:** Risk of bias assessment of studies.
**FIGURE S3:** Forest plot for complete resolution sub grouped by IU per injection.
**FIGURE S4:** Forest plot for partial resolution sub grouped by IU per injection.
**FIGURE S5:** Forest plot for no resolution sub grouped by IU per injection.
**FIGURE S6:** Forest plot for recurrence of wart sub grouped by IU per injection.
**FIGURE S7:** Forest plot for erythema sub grouped by IU per injection.
**FIGURE S8:** Forest plot for pain sub grouped by IU per injection.
**FIGURE S9:** Forest plot for swelling sub grouped by IU per injection.
**FIGURE S10:** Sensitivity analysis forest plot for complete resolution.
**FIGURE S11:** Sensitivity analysis forest plot for erythema.
**FIGURE S12:** Sensitivity analysis forest plot for pain.
**FIGURE S13:** Sensitivity analysis forest plot for swelling.
**FIGURE S14:** Funnel plots.
**TABLE S1:** Reported baseline wart types by each individual study.
**TABLE S2:** Previous treatment among groups reported by studies.
**TABLE S3:** Intervention protocols of individual studies.
**TABLE S4:** 2020 PRISMA checklist.

## Data Availability

The data that support the findings of this study are available from the corresponding author upon reasonable request.
