# Supplementary material for: Comparative Efficacy and Safety of Intralesional MMR Vaccine and Vitamin D3 in Managing Nongenital Warts: A Systematic Review and Meta‐Analysis
Source: J Cosmet Dermatol. 2026 Jan 8;25(1):e70618. doi: 10.1111/jocd.70618 (PMC12780659; doi:10.1111/jocd.70618)
Supplement: Supplementary file 1 — FIGURE S1: PRISMA flow diagram outlining the literature search process. FIGURE S2: Risk of bias assessment of studies. FIGURE S3: Forest plot for complete resolution sub grouped by IU per injection. FIGURE S4: Forest plot for partial resolution sub grouped by IU per injection. FIGURE S5: Forest plot for no resolution sub grouped by IU per injection. FIGURE S6: Forest plot for recurrence of wart sub grouped by IU per injection. FIGURE S7: Forest plot for erythema sub grouped by IU per injection. FIGURE S8: Forest plot for pain sub grouped by IU per injection. FIGURE S9: Forest plot for swelling sub grouped by IU per injection. FIGURE S10: Sensitivity analysis forest plot for complete resolution. FIGURE S11: Sensitivity analysis forest plot for erythema. FIGURE S12: Sensitivity analysis forest plot for pain. FIGURE S13: Sensitivity analysis forest plot for swelling. FIGURE S14: Funnel plots. TABLE S1: Reported baseline wart types by each individual study. TABLE S2: Previous treatment among groups reported by studies. TABLE S3: Intervention protocols of individual studies. TABLE S4: 2020 PRISMA checklist. [file JOCD-25-e70618-s001.docx]

| Table/Figure Number | Description |
| --- | --- |
| Figure S1: | PRISMA flow diagram outlining the literature search process. |
| Figure S2: | Risk of bias assessment of studies |
| Figure S3: | Forest Plot for Complete Resolution sub grouped by IU per injection |
| Figure S4: | Forest Plot for Partial Resolution sub grouped by IU per injection |
| Figure S5: | Forest Plot for no Resolution sub grouped by IU per injection |
| Figure S6: | Forest Plot for recurrence of wart sub grouped by IU per injection |
| Figure S7: | Forest Plot for erythema sub grouped by IU per injection |
| Figure S8: | Forest Plot for pain sub grouped by IU per injection |
| Figure S9: | Forest Plot for swelling sub grouped by IU per injection |
| Figure S10: | Sensitivity Analysis Forest Plot for Complete Resolution |
| Figure S11: | Sensitivity Analysis Forest Plot for Erythema |
| Figure S12: | Sensitivity Analysis Forest Plot for Pain |
| Figure S13: | Sensitivity Analysis Forest Plot for Swelling |
| Figure S14: | Funnel Plots |
| Table S1: | Reported baseline Wart types by each individual study |
| Table S2: | Previous treatment among groups reported by studies |
| Table S3: | Intervention protocols of individual studies. |
| Table S4: | 2020 PRISMA CHECKLIST |

**Supplementary Figure S1.** PRISMA flow diagram outlining the literature search process.














**Supplementary Figure S2:** Risk of bias assessment of studies

**
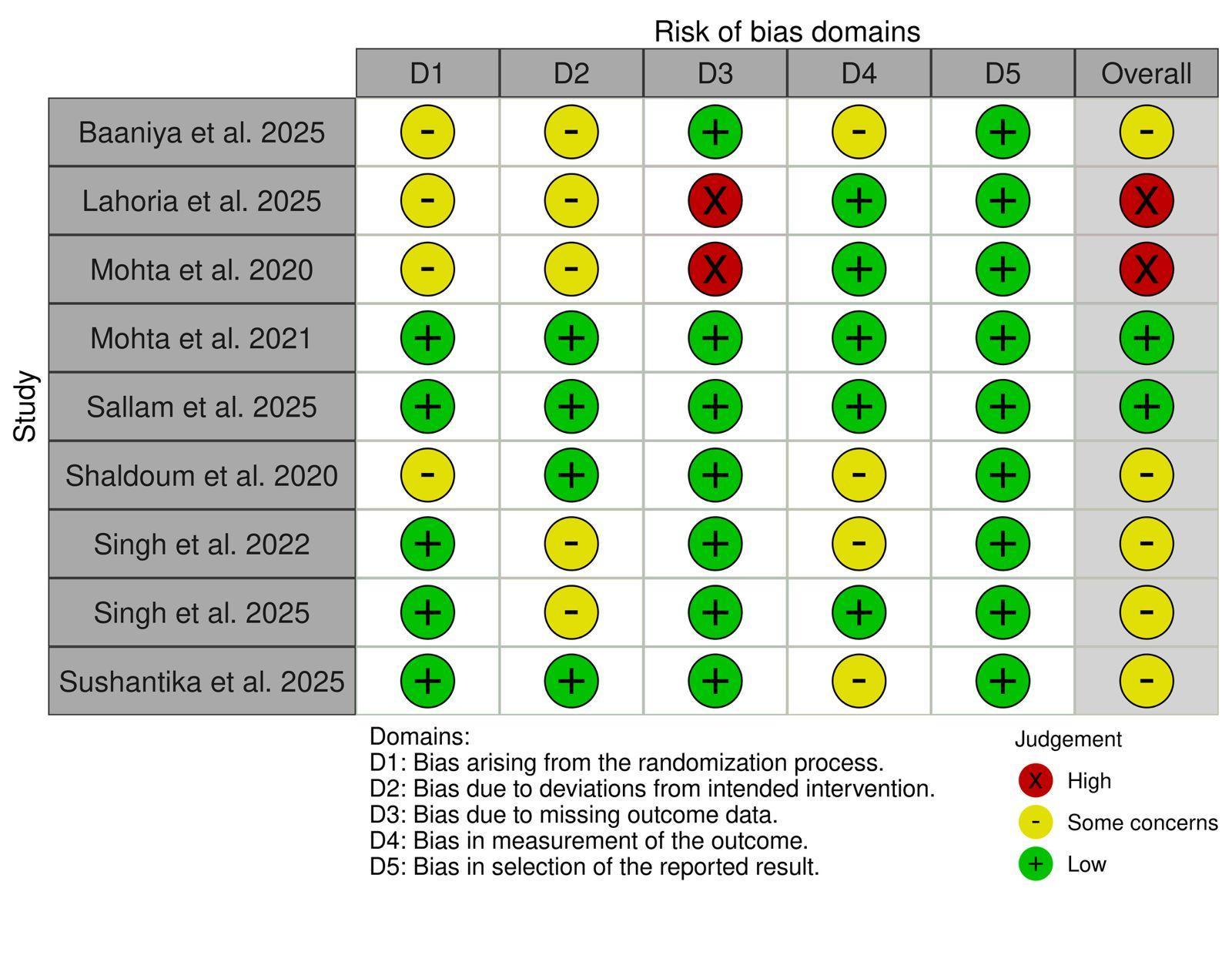
**

**Supplementary Figure S3:** Forest Plot for Complete Resolution sub grouped by IU per injection


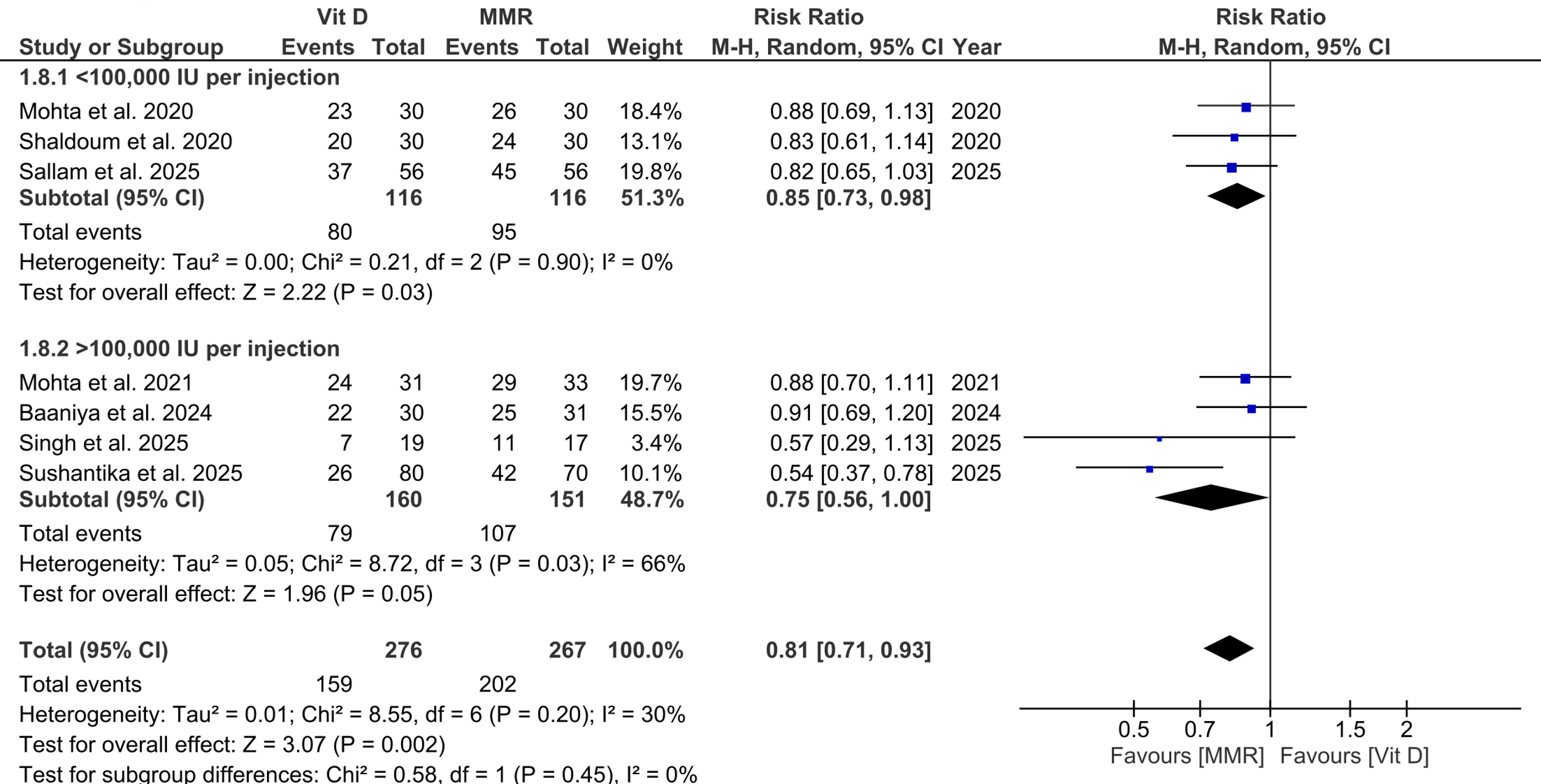


**Supplementary Figure S4**: Forest Plot for Partial Resolution sub grouped by IU per injection


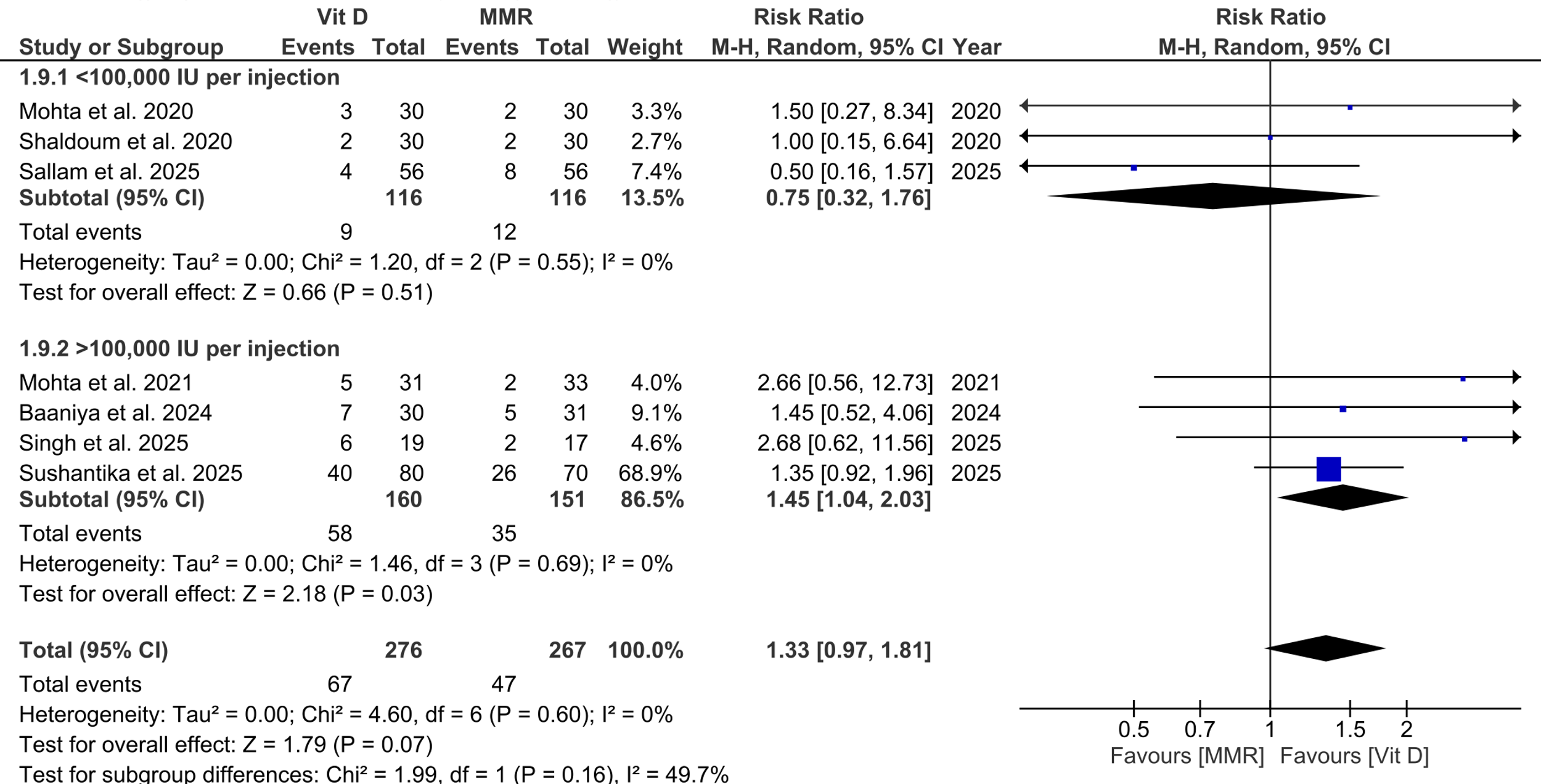


**Supplementary Figure S5**: Forest Plot for no Resolution sub grouped by IU per injection


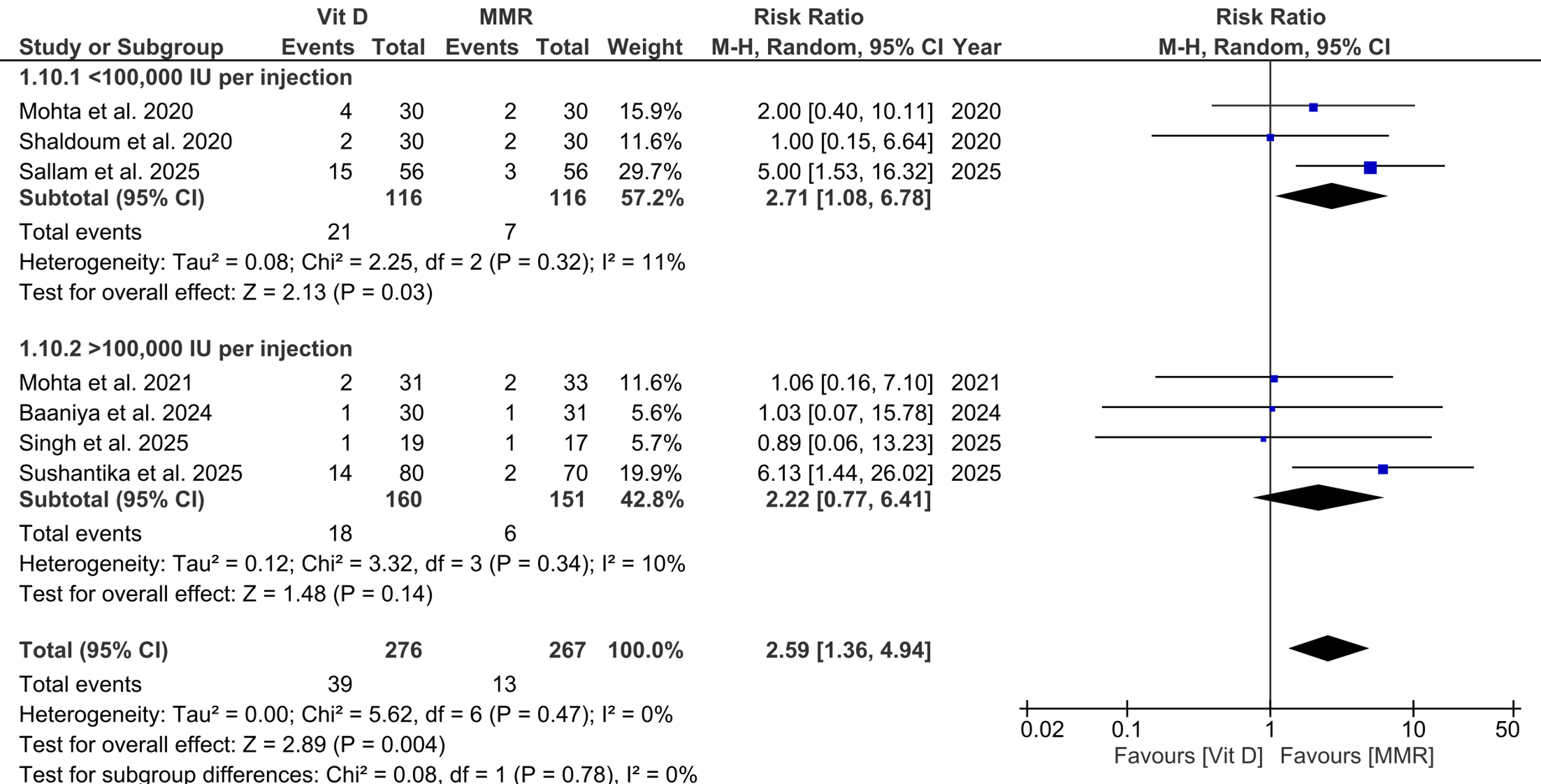


**Supplementary Figure S6:** Forest Plot for recurrence of wart sub grouped by IU per injection


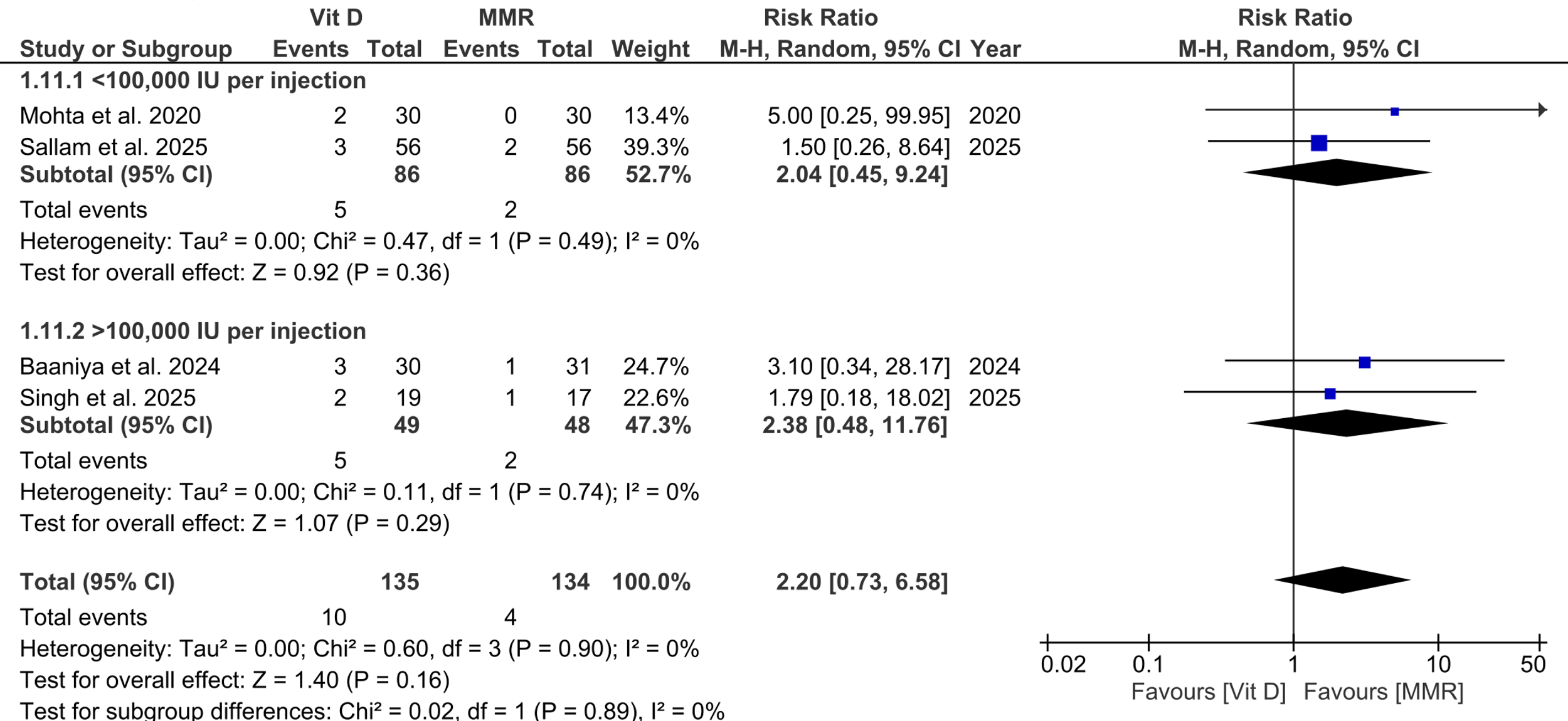


**Supplementary Figure S7:** Forest Plot for erythema sub grouped by IU per injection
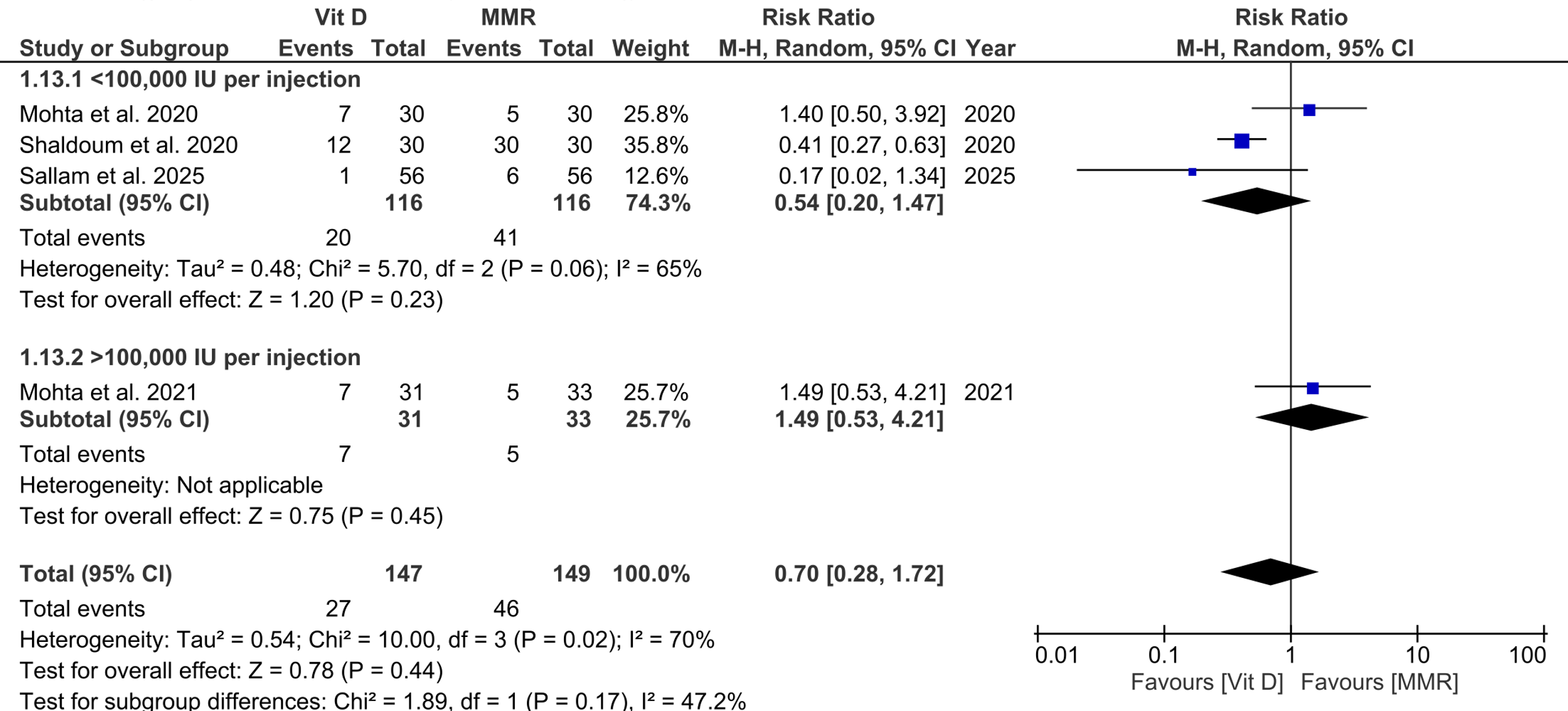


**Supplementary Figure S8:** Forest Plot for pain sub grouped by IU per injection
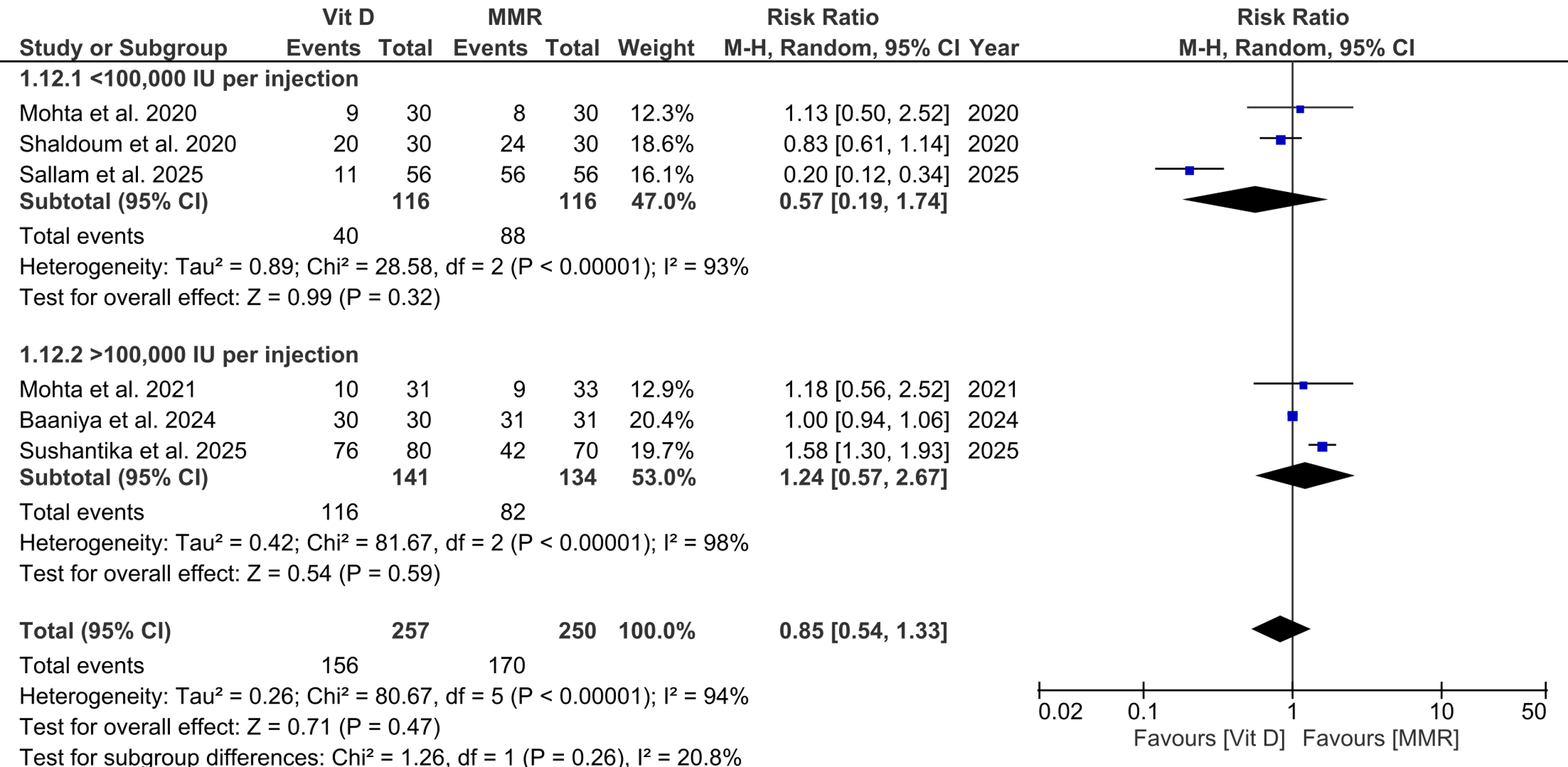


**Supplementary Figure S9:** Forest Plot for swelling sub grouped by IU per injection


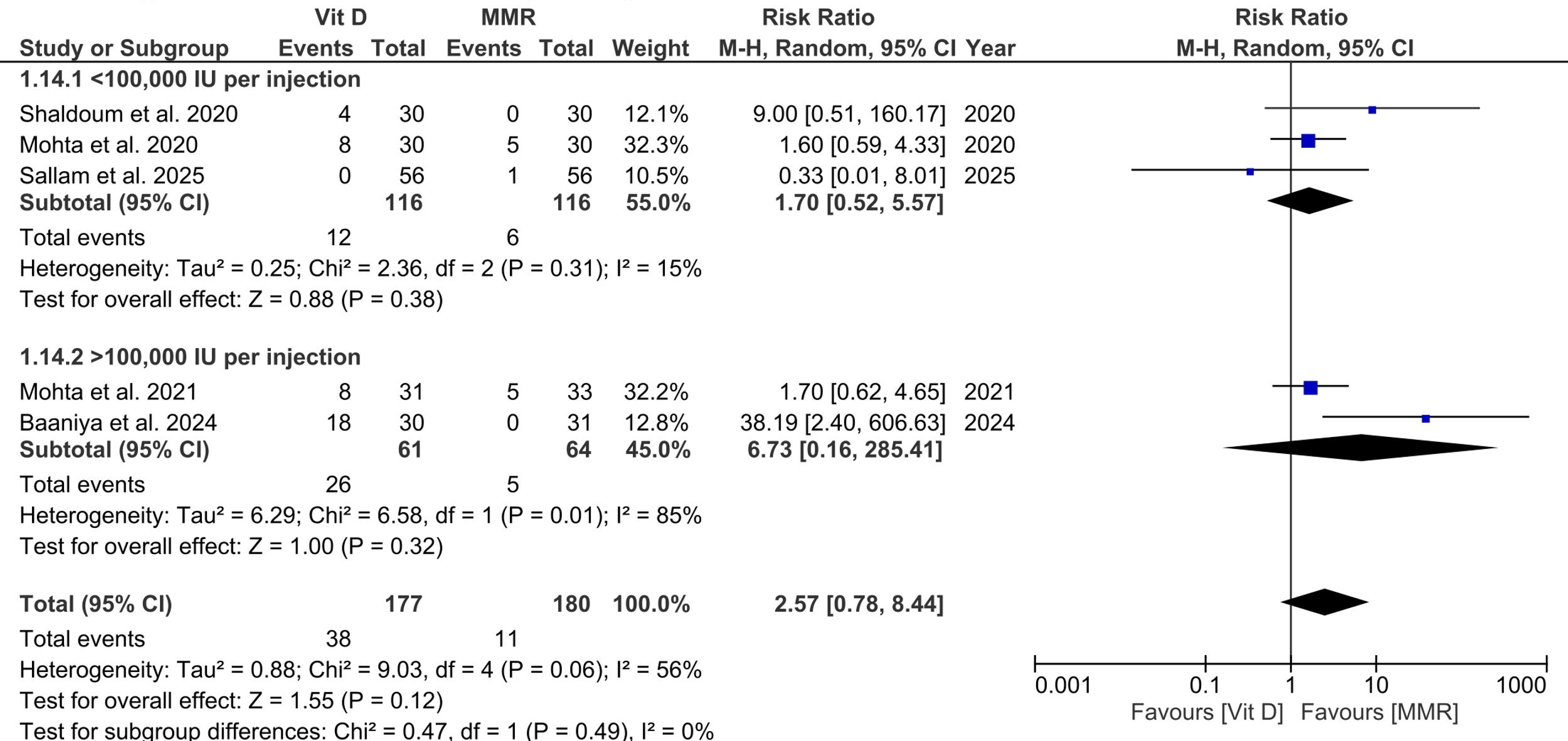


**Supplementary Figure S10:** Sensitivity Analysis Forest Plot for Complete Resolution


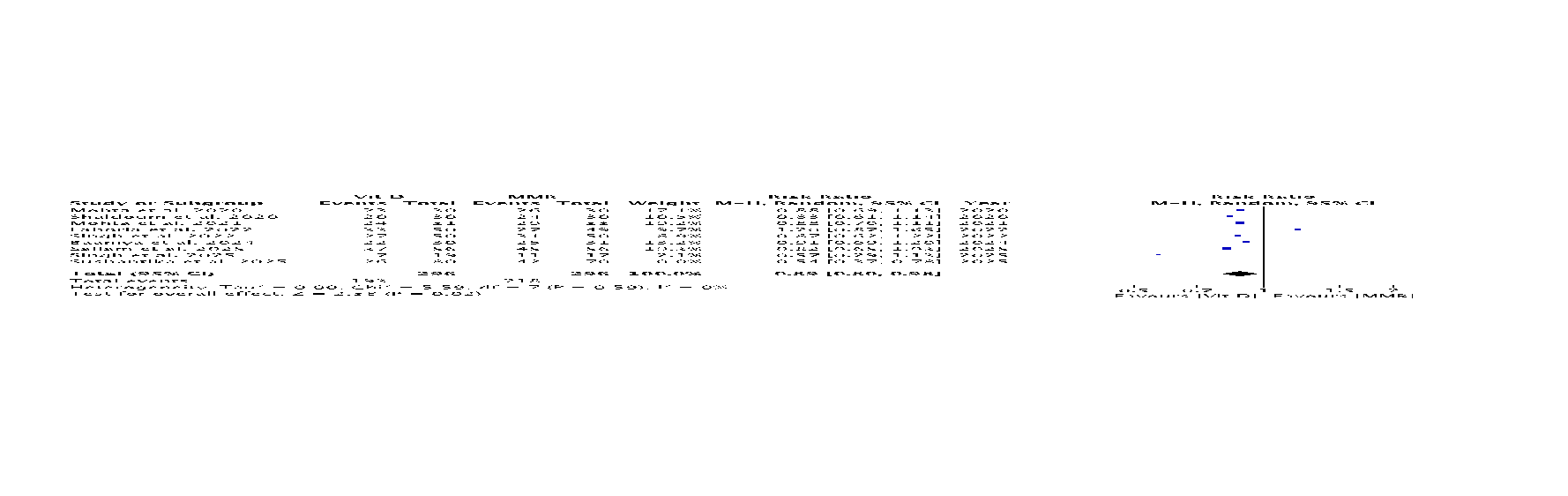


**Supplementary Figure S11:** Sensitivity Analysis Forest Plot for Erythema


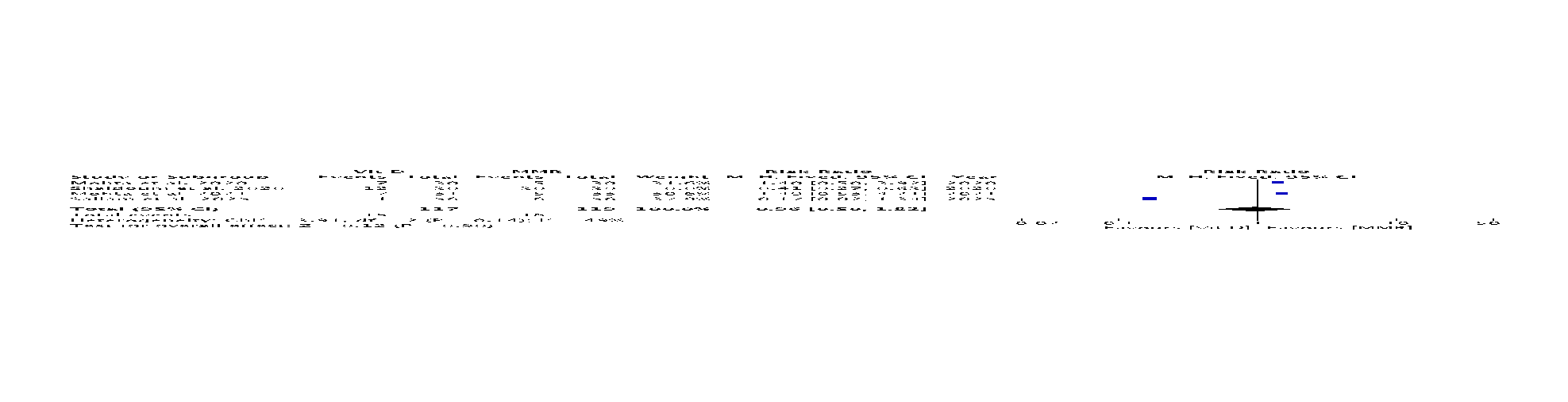


**Supplementary Figure S12:** Sensitivity Analysis Forest Plot for Pain


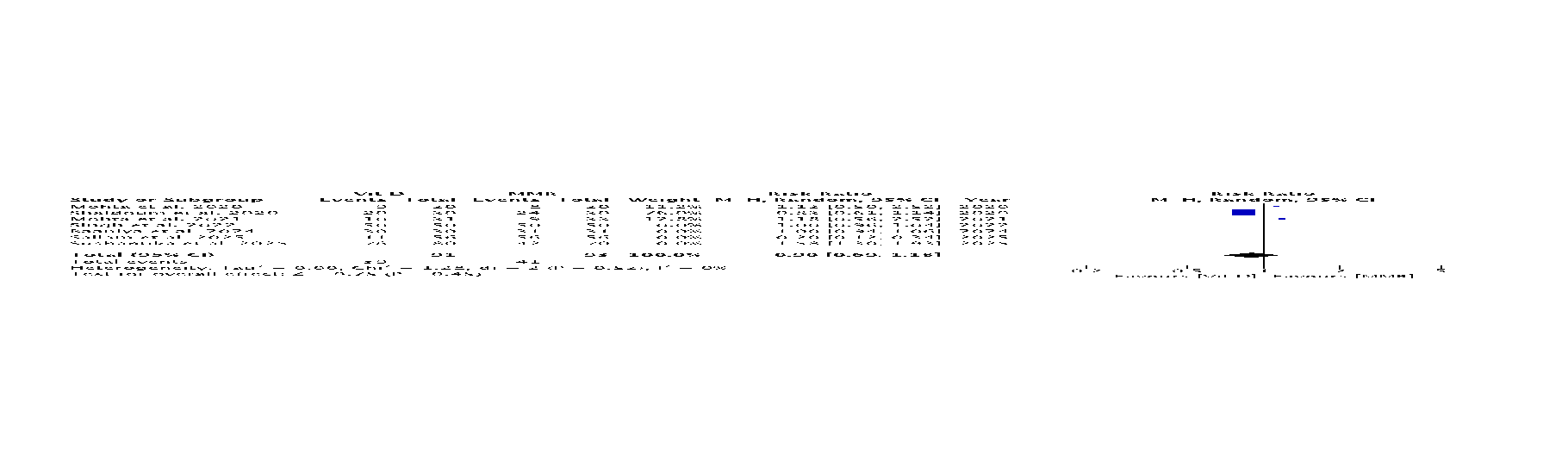


**Supplementary Figure S13:** Sensitivity Analysis Forest Plot for Swelling


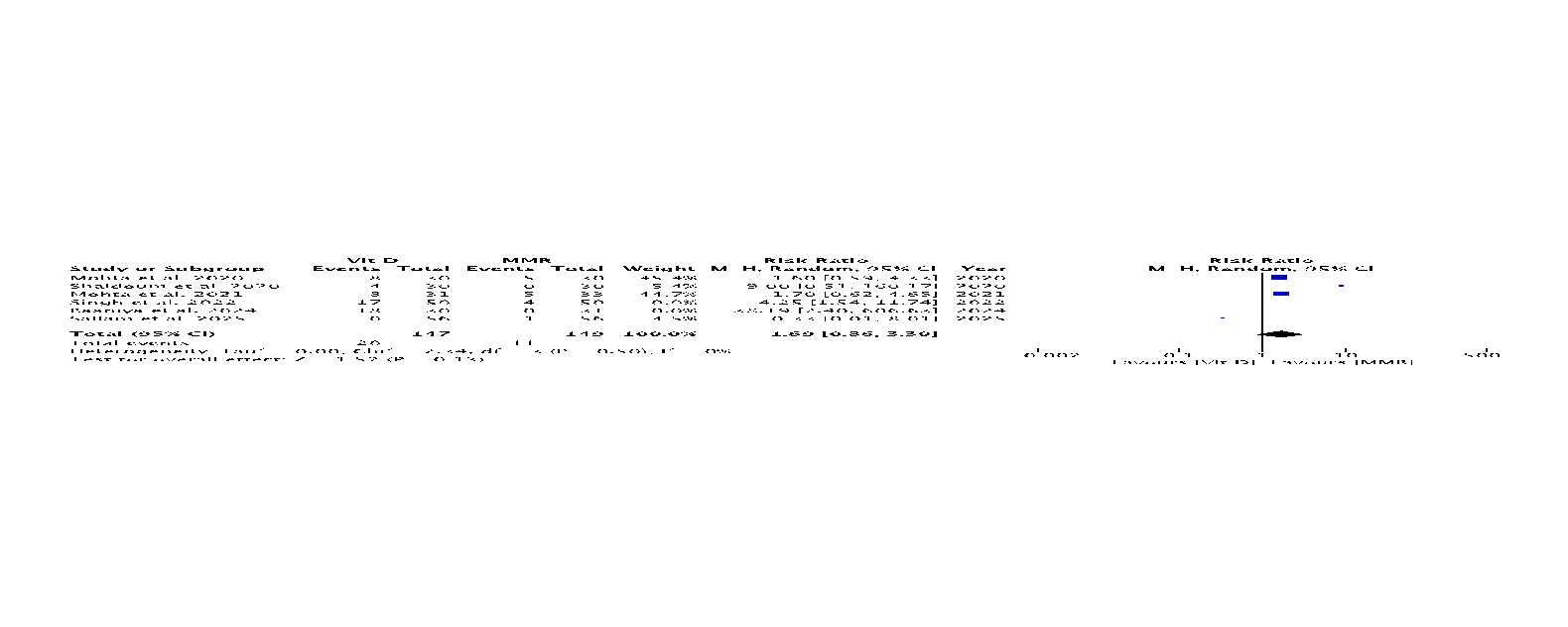


**Supplementary Figure S14:** Funnel Plots


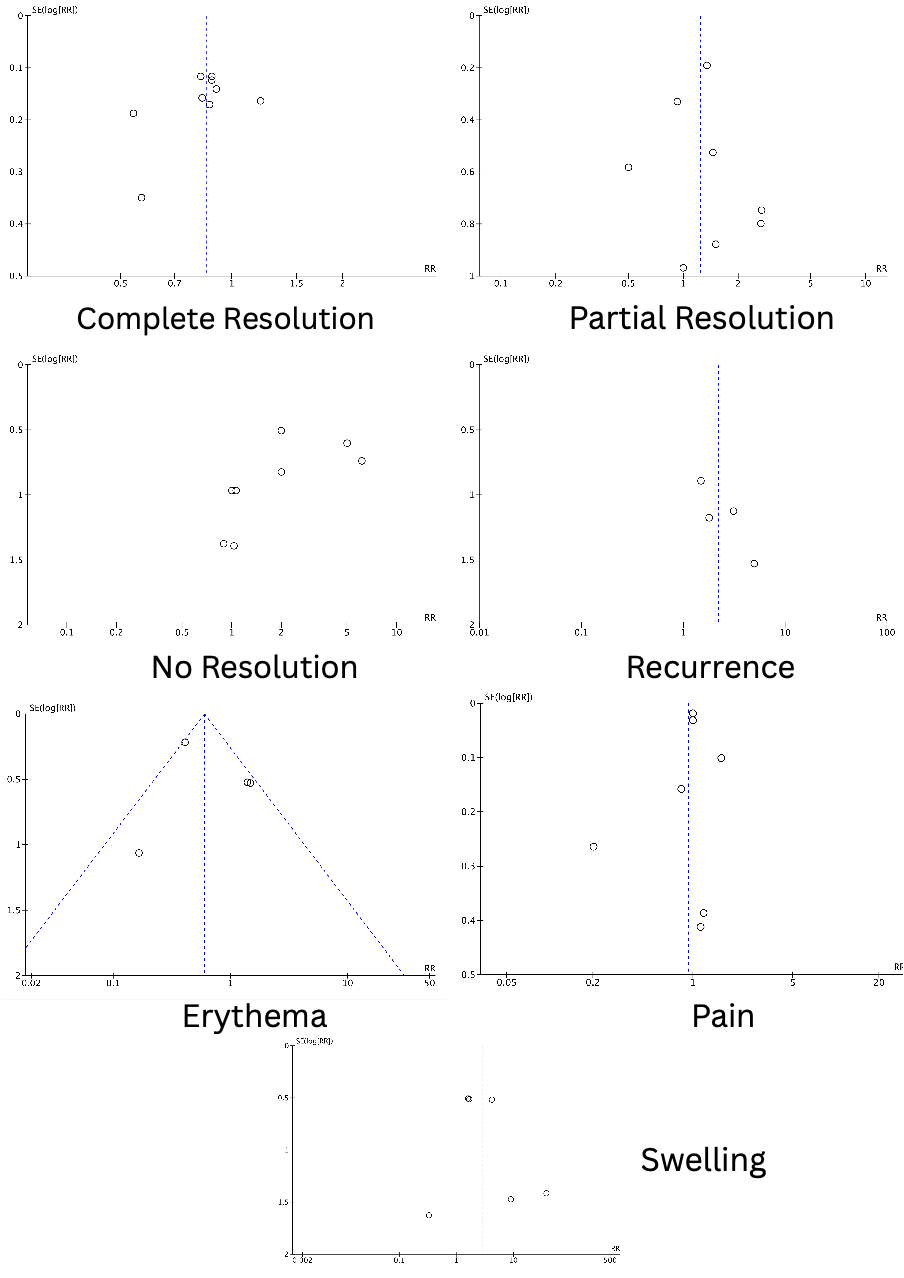


**Supplementary Table S1.** Reported baseline Wart types by each individual study

| Supplementary Table S1. | | | | | | | | | | | | | |
| --- | --- | --- | --- | --- | --- | --- | --- | --- | --- | --- | --- | --- | --- |
| Study | Verruca Vulgaris | | | Palmoplantaris warts | | Verruca Plana | | Genital warts | | Filiform warts | | Periungal warts | |
|  | MMR Group | Vitamin D Group | | MMR Group | Vitamin D Group | MMR Group | Vitamin D Group | MMR Group | Vitamin D Group | MMR Group | Vitamin D Group | MMR Group | Vitamin D Group |
| Shaldoum et al. 2020 | 10 | | 12 | 14 | 12 | 0 | 0 | 0 | 0 | 0 | 0 | 6 | 6 |
| Mohta et al. 2021 | 12 | | 13 | 11 | 8 | 7 | 9 | 0 | 0 | 0 | 0 | 3 | 1 |
| Singh et al. 2022 | 12 | | 10 | 22 | 22 | 8 | 7 | 2 | 2 | 2 | 4 | 4 | 5 |
| Lahoria et al. 2022 | 26 | | 20 | 9 | 13 | 9 | 10 | 0 | 0 | 4 | 7 | 1 | 0 |
| Sushantika et al. 2025 | 12 | | 16 | 43 | 42 | 0 | 0 | 6 | 7 | 9 | 15 | 0 | 0 |
| Baaniya et al. 2025 | 17 | | 18 | 22 | 19 | 0 | 0 | 0 | 0 | 0 | 0 | 6 | 6 |
| Sallam et al. 2025 | 11 | | 22 | 26 | 23 | 11 | 11 | 0 | 0 | 0 | 0 | 0 | 0 |
| *Note*. Data are n. Types of warts was not reported in the studies Mohta et al. 2020 and Singh et al. 2025. | | | | | | | | | | | | | |

**Supplementary Table S2**. Previous treatment among groups reported by studies

| Supplementary Table S2. | | | | |
| --- | --- | --- | --- | --- |
| Study | MMR Group, n (%) | | Vitamin D Group, n (%) | |
|  | Yes | No | Yes | No |
| Shaldoum et al. 2020 | 20 (66.7) | 10 (33.3) | 16 (53.3) | 14 (46.7) |
| Lahoria et al. 2022 | 2 (4.1) | 47 (95.9) | 3 (6.0) | 44 (88.0) |
| Baaniya et al. 2025 | 10 (32.3) | 21 (67.7) | 11 (36.7) | 19 (63.4) |
| Sallam et al. 2025 | 37 (66.1) | 19 (33.9) | 30 (53.6) | 26 (46.4) |
| *Note.* Data are n (%). All studies excluded participants with prior treatment in the last 4 weeks, except Sushantika et al., which used a 3-week interval and Sallam et al., which applied a 3-month interval. In Baaniya et al. 2025: MMR Group - 2 traditional treatment, 8 medical treatment; Vitamin D Group - 1 traditional treatment, 10 medical treatment. Previous treatment among groups was only reported in detail in above 4 studies. | | | | |

**Supplementary Table S3.** Intervention protocols of individual studies.

| Supplementary Table S3. | | | | | | | | |  |
| --- | --- | --- | --- | --- | --- | --- | --- | --- | --- |
| Study | MMR Vaccine | | | | |  | MMR Vaccine and Vitamin D3 | |  |
|  | Vaccine Brand | Manufacturer/ Country | Strain content/ Formulation | Dose injected per lesion, (mL) | Site |  | Dosing interval, weeks | Maximum treatment sessions, n |  |
| Shaldoum et al. 2020 | VACSERA | Egypt | freeze-dried, 0.5ml vial | 0.3 | into largest wart |  | 3 | 6 |  |
| Mohta et al. 2020 | - | - | - | 0.1 | into largest wart |  | 4 | 3 | |
| Mohta et al. 2021 | TRESIVAC | Serum Institute of India LTD., Pune, Maharashtra, India | freeze-dried | 0.2-0.3 | into largest wart |  | 2 | 4 |  |
| Singh et al. 2022 | - | - | - | 0.3 | into largest wart |  | 2 | 3 |  |
| Lahoria et al. 2022 | - | - | - | 0.5 | into largest wart |  | 2 | - |  |
| Sushantika et al. 2025 | TRESIVAC | Serum Institute of India LTD., Pune, Maharashtra, India | freeze-dried, 0.5ml, rubella vaccine (1000.0 Ccid) + mumps vaccine (5000.0 Ccid) + measles vaccines (1000.0 Ccid) | 0.3 + 0.1 | into largest wart + other warts |  | 3 | 6 |  |
| Singh et al. 2025 | TRESIVAC | Serum Institute of India LTD., Pune, Maharashtra, India | freeze-dried | 0.5 | into largest wart |  | 3 | 3 |  |
| Baaniya et al. 2025 | - | - | - | - | - |  | 3 | 5 |  |
| Sallam et al. 2025 | VACSERA | Egypt | freeze-dried, 0.5ml vial | 0.3 | into largest wart |  | 2 | 5 |  |
| *Note. -* = not reported by the study. All studies used MMR vaccine diluted in 0.5ml distilled water (dilution information not reported in studies Singh et al. 2022 and Lahoria et al. 2022). Studies Shaldoum et al. 2020, Sushantika et al. 2025 and Baaniya et al. 2025 injected 5 warts per session. | | | | | | | | |  |

**Supplementary Table S4.** 2020 PRISMA Checklist

| **Topic** | **No.** | **Item** | **Location where item is reported** |
| --- | --- | --- | --- |
| **TITLE** |  |  |  |
| **Title** | **1** | **Identify the report as a systematic review.** | **Page 1** |
| **ABSTRACT** |  |  |  |
| **Abstract** | **2** | **See the PRISMA 2020 for Abstracts checklist** |  |
| **INTRODUCTION** |  |  |  |
| **Rationale** | **3** | **Describe the rationale for the review in the context of existing knowledge.** | **Introduction Paragraph 5** |
| **Objectives** | **4** | **Provide an explicit statement of the objective(s) or question(s) the review addresses.** | **Introduction Paragraph 5** |
| **METHODS** |  |  |  |
| **Eligibility criteria** | **5** | **Specify the inclusion and exclusion criteria for the review and how studies were grouped for the syntheses.** | **Methods 2.1 "Eligibility Criteria"** |
| **Information sources** | **6** | **Specify all databases, registers, websites, organisations, reference lists and other sources searched or consulted to identify studies. Specify the date when each source was last searched or consulted.** | **Methods 2.2 "Search Strategy" Paragraph 1** |
| **Search strategy** | **7** | **Present the full search strategies for all databases, registers and websites, including any filters and limits used.** | **Methods 2.2 "Search Strategy" Paragraph 1** |
| **Selection process** | **8** | **Specify the methods used to decide whether a study met the inclusion criteria of the review, including how many reviewers screened each record and each report retrieved, whether they worked independently, and if applicable, details of automation tools used in the process.** | **Methods 2.2 "Search Strategy" Paragraph 2** |
| **Data collection process** | **9** | **Specify the methods used to collect data from reports, including how many reviewers collected data from each report, whether they worked independently, any processes for obtaining or confirming data from study investigators, and if applicable, details of automation tools used in the process.** | **Methods 2.4 "Data Extraction and Quality Assessment" Paragraph 1 and 2** |
| **Data items** | **10a** | **List and define all outcomes for which data were sought. Specify whether all results that were compatible with each outcome domain in each study were sought (e.g. for all measures, time points, analyses), and if not, the methods used to decide which results to collect.** | **Methods 2.3 "Endpoints"** |
|  | **10b** | **List and define all other variables for which data were sought (e.g. participant and intervention characteristics, funding sources). Describe any assumptions made about any missing or unclear information.** | **Methods 2.3 "Endpoints"** |
| **Study risk of bias assessment** | **11** | **Specify the methods used to assess risk of bias in the included studies, including details of the tool(s) used, how many reviewers assessed each study and whether they worked independently, and if applicable, details of automation tools used in the process.** | **Methods 2.4 "Data Extraction and Quality Assessment" Paragraph 1 and 2** |
| **Effect measures** | **12** | **Specify for each outcome the effect measure(s) (e.g. risk ratio, mean difference) used in the synthesis or presentation of results.** | **Methods 2.5 "Statistical Analysis"** |
| **Synthesis methods** | **13a** | **Describe the processes used to decide which studies were eligible for each synthesis (e.g. tabulating the study intervention characteristics and comparing against the planned groups for each synthesis (item 5)).** | **Methods 2.5 "Statistical Analysis"** |
|  | **13b** | **Describe any methods required to prepare the data for presentation or synthesis, such as handling of missing summary statistics, or data conversions.** | **Methods 2.5 "Statistical Analysis"** |
|  | **13c** | **Describe any methods used to tabulate or visually display results of individual studies and syntheses.** | **Methods 2.5 "Statistical Analysis"** |
|  | **13d** | **Describe any methods used to synthesize results and provide a rationale for the choice(s). If meta-analysis was performed, describe the model(s), method(s) to identify the presence and extent of statistical heterogeneity, and software package(s) used.** | **Methods 2.5 "Statistical Analysis"** |
|  | **13e** | **Describe any methods used to explore possible causes of heterogeneity among study results (e.g. subgroup analysis, meta-regression).** | **Methods 2.5 "Statistical Analysis"** |
|  | **13f** | **Describe any sensitivity analyses conducted to assess robustness of the synthesized results.** | **Methods 2.5 "Statistical Analysis"** |
| **Reporting bias assessment** | **14** | **Describe any methods used to assess risk of bias due to missing results in a synthesis (arising from reporting biases).** | **Methods 2.4 "Data Extraction and Quality Assessment" Paragraph 1 and 2** |
| **Certainty assessment** | **15** | **Describe any methods used to assess certainty (or confidence) in the body of evidence for an outcome.** | **Methods 2.4 "Data Extraction and Quality Assessment" Paragraph 1 and 2** |
| **RESULTS** |  |  |  |
| **Study selection** | **16a** | **Describe the results of the search and selection process, from the number of records identified in the search to the number of studies included in the review, ideally using a flow diagram.** | **Results 3.1 "Study Selection and Baseline Characteristics"** |
|  | **16b** | **Cite studies that might appear to meet the inclusion criteria, but which were excluded, and explain why they were excluded.** | **Results 3.1 "Study Selection and Baseline Characteristics"** |
| **Study characteristics** | **17** | **Cite each included study and present its characteristics.** | **Results 3.1 "Study Selection and Baseline Characteristics"** |
| **Risk of bias in studies** | **18** | **Present assessments of risk of bias for each included study.** | **Results 3.1 "Study Selection and Baseline Characteristics"** |
| **Results of individual studies** | **19** | **For all outcomes, present, for each study: (a) summary statistics for each group (where appropriate) and (b) an effect estimate and its precision (e.g. confidence/credible interval), ideally using structured tables or plots.** | **Results 3.2 "Pooled Analysis of All Studies"** |
| **Results of syntheses** | **20a** | **For each synthesis, briefly summarise the characteristics and risk of bias among contributing studies.** | **Results 3.2 "Pooled Analysis of All Studies"** |
|  | **20b** | **Present results of all statistical syntheses conducted. If meta-analysis was done, present for each the summary estimate and its precision (e.g. confidence/credible interval) and measures of statistical heterogeneity. If comparing groups, describe the direction of the effect.** | **Results 3.2 "Pooled Analysis of All Studies"** |
|  | **20c** | **Present results of all investigations of possible causes of heterogeneity among study results.** | **Results 3.2 "Pooled Analysis of All Studies"** |
|  | **20d** | **Present results of all sensitivity analyses conducted to assess the robustness of the synthesized results.** | **Results 3.2 "Pooled Analysis of All Studies"** |
| **Reporting biases** | **21** | **Present assessments of risk of bias due to missing results (arising from reporting biases) for each synthesis assessed.** | **Results 3.1 "Study Selection and Baseline Characteristics"** |
| **Certainty of evidence** | **22** | **Present assessments of certainty (or confidence) in the body of evidence for each outcome assessed.** | **Results 3.1 "Study Selection and Baseline Characteristics"** |
| **DISCUSSION** |  |  |  |
| **Discussion** | **23a** | **Provide a general interpretation of the results in the context of other evidence.** | **Discussion paragraph 1-13** |
|  | **23b** | **Discuss any limitations of the evidence included in the review.** | **Limitations and Conclusion** |
|  | **23c** | **Discuss any limitations of the review processes used.** | **Limitations and Conclusion** |
|  | **23d** | **Discuss implications of the results for practice, policy, and future research.** | **Discussion paragraph 14** |
| **OTHER INFORMATION** |  |  |  |
| **Registration and protocol** | **24a** | **Provide registration information for the review, including register name and registration number, or state that the review was not registered.** | **Methods paragraph 1** |
|  | **24b** | **Indicate where the review protocol can be accessed, or state that a protocol was not prepared.** | **Methods paragraph 1** |
|  | **24c** | **Describe and explain any amendments to information provided at registration or in the protocol.** | **Methods paragraph 1** |
| **Support** | **25** | **Describe sources of financial or non-financial support for the review, and the role of the funders or sponsors in the review.** | **Declarations section** |
| **Competing interests** | **26** | **Declare any competing interests of review authors.** | **Declarations section** |
| **Availability of data, code and other materials** | **27** | **Report which of the following are publicly available and where they can be found: template data collection forms; data extracted from included studies; data used for all analyses; analytic code; any other materials used in the review.** | **Declarations section** |

***From:* Page MJ, McKenzie JE, Bossuyt PM, Boutron I, Hoffmann TC, Mulrow CD, et al. The PRISMA 2020 statement: an updated guideline for reporting systematic reviews. MetaArXiv. 2020, September 14. DOI: 10.31222/osf.io/v7gm2. For more information, visit: www.prisma-statement.org**
